# Development and Temporal Validation of a Multinomial Prediction Model for Phenotypes of Undiagnosed Hypertension in Peru: A Population-Based Study

**DOI:** 10.3390/medsci14020224

**Published:** 2026-04-29

**Authors:** Víctor Juan Vera-Ponce, Jhosmer Ballena-Caicedo, Holly Estrella Delgado-Toro, Fiorella E. Zuzunaga-Montoya, Julio César Bautista Zuta, Rossmery Leonor Poemape Mestanza

**Affiliations:** 1 Facultad de Medicina (FAMED), Universidad Nacional Toribio Rodríguez de Mendoza de Amazonas (UNTRM), Chachapoyas 01001, Peru; 7330178022@untrm.edu.pe (J.B.-C.); 7694394622@untrm.edu.pe (H.E.D.-T.); fiorellazuzunaga@gmail.com (F.E.Z.-M.); julio.bautista@untrm.edu.pe (J.C.B.Z.); rossmerylpm@gmail.com (R.L.P.M.); 2 Hospital Regional Virgen de Fátima, Chachapoyas 01001, Peru

**Keywords:** hypertension, undiagnosed hypertension, prediction model, multinomial logistic regression, blood pressure phenotypes, Peru

## Abstract

**Background/Objectives:** Early diagnosis of hypertension (HTN) is critical, but most screening models do not simultaneously distinguish phenotypes based on systolic or diastolic patterns. We developed and temporally validated a multinomial model to predict normotension and three phenotypes of undiagnosed hypertension in Peru. **Methods:** We used ENDES 2017–2019 for development (final analytic *n* = 62,091) and ENDES 2021–2024 for temporal validation (final analytic *n* = 77,372), excluding 2020 due to COVID-19 disruptions. We included adults aged ≥18 years without self-reported HTN. The outcome was classified as normotension, isolated diastolic hypertension (IDH), isolated systolic hypertension (ISH), or systolic–diastolic hypertension (SDH). Eight nonlaboratory predictors were used: age, BMI, sex, residential altitude, smoking, alcohol consumption, vegetable intake, and fruit intake. **Results:** The model achieved an AUC of 0.789 (95% CI: 0.783–0.795) in training and 0.776 (95% CI: 0.770–0.781) in temporal validation. The prevalence of undiagnosed hypertension was 11.6% in the training set and 12.6% in the validation set. At a prespecified cutoff of 0.1004, sensitivity and specificity were 79.0% and 63.2% in training and 78.7% and 60.9% in validation, respectively (NPV 95.8% and 95.2%). Decision curve and clinical impact analyses suggested a positive net benefit and plausible referral volumes across a range of thresholds. **Conclusions:** This model could help prioritize confirmatory blood pressure measurements in resource-limited settings.

## 1. Introduction

Hypertension (HTN) is one of the leading modifiable risk factors for cardiovascular disease and premature mortality. In 2019, an estimated 1.28 billion adults aged 30–79 years were living with hypertension, and global control rates remained low [1]. The burden is disproportionately concentrated in low- and middle-income countries, where gaps persist in diagnosis, treatment, and control compared with high-income countries [1,2,3].

Despite the availability of blood pressure measurement and cost-effective treatments in primary care, many individuals with hypertension remain undiagnosed or uncontrolled, especially in resource-limited settings [1]. Multinational studies in low- and middle-income countries show substantial losses across the care cascade—from having previously been measured to diagnosis, treatment, and control—and marked inequities by socioeconomic status and place of residence [4].

Moreover, hypertension is heterogeneous and can be classified, according to the pattern of systolic and/or diastolic elevation, into isolated systolic hypertension (ISH), isolated diastolic hypertension (IDH), and systolic–diastolic hypertension (SDH). These subtypes show distinct distributions by age and sex, may change over time, and have been associated with differential cardiovascular risks, supporting the value of considering the systolic/diastolic pattern when planning clinical assessment and follow-up [5,6,7]. Globally, prevalence estimates for each subtype have been reported, with substantial heterogeneity across populations [8]. However, in screening and in many predictive approaches, this heterogeneity is often obscured when hypertension is treated as a binary outcome.

In this context, tiered risk-based strategies and prediction tools using simple variables could complement opportunistic screening by focusing on active case finding and prioritizing confirmatory measurement, without replacing it [9]. In Peru, where hypertension is a major public health problem, scalable approaches are needed to strengthen the identification of people with probable undiagnosed hypertension and to guide prioritization efforts at the primary care level [10]. Most existing hypertension prediction models are cross-sectional, binary, and developed in Asian populations. Incorporating laboratory biomarkers provides only a marginal benefit over models using clinical variables alone [11]. To our knowledge, no models developed and validated in Latin American populations simultaneously estimate the probabilities of normotension and of ISH, IDH, and SDH among individuals without a prior diagnosis.

Therefore, we developed and temporally validated a multinomial model to predict normotension and phenotypes of undiagnosed hypertension in Peruvian adults, using only nonlaboratory variables obtainable through interviews and simple measurements, to support the prioritization of confirmatory blood pressure measurement and clinical assessment in primary care. This model is not intended to replace blood pressure measurement but to support tiered active case-finding and prioritization where universal, repeated coverage is operationally infeasible.

## 2. Materials and Methods

### 2.1. Study Design and Data Source

We developed and temporally validated a prediction model, without laboratory tests, to identify undiagnosed hypertension and its blood pressure phenotypes in Peruvian adults. For model development, we followed the recommendations of the TRIPOD + AI (Transparent Reporting of a multivariable prediction model for Individual Prognosis or Diagnosis + AI) guidelines, updated in 2024 [12]. The completed TRIPOD + AI checklist is provided in Supplementary Table S1.

#### Data Source

We used data from Peru’s Demographic and Family Health Survey (ENDES), a nationally representative population-based cross-sectional survey conducted annually by the National Institute of Statistics and Informatics (INEI). ENDES uses a two-stage, stratified, cluster sampling design with probabilistic selection of households in urban and rural areas across the country’s 24 departments and the Constitutional Province of Callao. The adult health module includes anthropometric and blood pressure measurements performed by trained personnel in accordance with standardized protocols [13].

### 2.2. Datasets and Eligibility Criteria

Two temporally independent datasets were defined: a training dataset (ENDES 2017–2019) and a temporal validation dataset (ENDES 2021–2024).

We excluded: (1) participants with a self-reported prior hypertension diagnosis; (2) individuals younger than 18 years; (3) records with missing data in the outcome or any predictor; and (4) biologically implausible blood pressure and body mass index (BMI) values, according to the prespecified filters described below. Missing data were handled using a complete-case approach; no imputation was performed. This decision was supported by the low proportion of exclusions due to missingness or implausible BMI in both datasets (Figure S1); nonetheless, selection bias cannot be ruled out.

### 2.3. Blood Pressure Measurement and Outcome Definition

Blood pressure was measured by trained ENDES personnel using calibrated digital sphygmomanometers (OMRON HEM-713; OMRON Healthcare Co., Ltd., Muko, Kyoto, Japan). Three consecutive measurements were taken on the left arm after at least five minutes of rest, with at least one-minute intervals between measurements. The mean of the second and third measurements was used as each participant’s systolic blood pressure (SBP) and diastolic blood pressure (DBP), following the standardized protocol described in the ENDES methodological guide. For quality control, SBP values <60 or ≥300 mmHg and DBP values <30 or ≥200 mmHg were considered implausible and recoded as missing.

The multinomial outcome comprised four mutually exclusive categories, defined using clinical thresholds of 140/90 mmHg [5,14,15]: normotension (SBP < 140 and DBP < 90), isolated diastolic hypertension (IDH: SBP < 140 and DBP ≥ 90), isolated systolic hypertension (ISH: SBP ≥ 140 and DBP < 90), and systolic–diastolic hypertension (SDH: SBP ≥140 and DBP ≥ 90). We treated these categories as blood pressure phenotypes, reflecting the systolic and/or diastolic pattern of the measurement rather than a direct hemodynamic characterization. To evaluate overall performance, we additionally defined a binary composite outcome of any undiagnosed hypertension (IDH, ISH, or SDH) versus normotension. Because ENDES measures blood pressure during a single visit, this outcome represents elevated blood pressure compatible with undiagnosed hypertension rather than a clinical diagnosis confirmed across visits.

We adopted a 140/90 mmHg threshold to define undiagnosed hypertension rather than a lower 130/80 mmHg threshold used in some diagnostic frameworks [14]. This choice reflects the continued use of 140/90 mmHg in major international hypertension guidance [15], the fact that ENDES measurements are obtained during a single household visit without confirmatory readings on subsequent days or ambulatory monitoring, and the need to preserve comparability with prior Peruvian and Latin American epidemiologic studies [10,16,17]. In this context, a lower threshold would increase the proportion of false positives due to inherent measurement variability and reduce the model’s utility as a screening tool.

### 2.4. Candidate Predictors

We prespecified eight nonlaboratory predictors routinely available in ENDES, selected for clinical plausibility and feasibility for community screening. We prioritized variables obtainable through structured interviews and basic anthropometry, without laboratory tests or specialized instruments other than a scale and a stadiometer.

The two continuous variables were age (completed years at the time of the interview) and body mass index (BMI, kg/m^2^), calculated from weight and height measured by ENDES personnel using calibrated equipment. BMI was considered biologically implausible if <10 or ≥60 kg/m^2^ and was recoded as missing.

The six categorical variables and their operationalization in ENDES were: sex (female, male; variable HV104); residential altitude based on variable HV040 from the household questionnaire, categorized as <1500 m, 1500–2499 m, 2500–3499 m, and ≥3500 m; smoking status, defined from questions on lifetime tobacco use and current smoking (variables QS700–QS707), classified as never smoker, former smoker (ever smoked but not currently), current smoker (smokes but not daily), and daily smoker (smokes every day); alcohol consumption, derived from questions on frequency and quantity of alcohol consumption in the past 30 days (variables QS600–QS614), categorized as none (no consumption in the past 30 days), moderate consumption (consumption without risky episodes), and risky consumption (five or more standard drinks on one occasion at least once in the past 30 days) [18]; and vegetable intake and fruit intake, treated as binary variables (yes/no). Both variables were derived from ENDES questions on the number of portions consumed per day: “How many units, slices, or bunches of fruit did you eat per day?” (variable QS214C) and “How many slices of vegetables did you eat per day?” (variable QS220CV). Fruit intake was classified as affirmative if the participant reported three or more portions per day, and vegetable intake was classified as affirmative if two or more portions per day were reported [19]. Participants with missing values for these dietary variables were excluded from the complete-case analysis.

We did not include self-identified ethnicity or the wealth index, despite their availability in ENDES. Self-identified ethnicity had a high proportion of missing data that differed substantially between the training and validation datasets, potentially introducing differential selection bias if the analytic sample were restricted to complete cases. The wealth index is a household-level construct derived from assets and housing characteristics and is not readily communicable to individuals or directly operationalizable in a community screening tool. Excluding it prioritized implementability, although doing so may reduce predictive performance or limit equity assessment; future sensitivity analyses should examine models that include socioeconomic variables.

### 2.5. Model Development

We fitted a multinomial logistic regression model with four response categories (normotension as the reference category, with IDH, ISH, and SDH as the comparison categories) using the multinom function from the nnet package in R, which simultaneously estimates three logit equations—one for each phenotype versus the reference category—by maximizing the joint likelihood with up to 500 iterations. The model therefore estimated, for each participant, four mutually exclusive and exhaustive probabilities that sum to one.

All eight predictors were entered into the model without variable selection, as they were prespecified based on the available evidence. We did not apply automated reduction techniques (stepwise selection, LASSO) or assess the incremental contribution of individual predictors to the AUC; prespecifying the full set of predictors reduces the risk of overfitting from data-driven selection.

Age and BMI were modeled using natural cubic splines generated with the ns function from the splines package in R, each with 2 degrees of freedom. This parameterization places one internal knot at the median of the training distribution and boundary knots at the observed minimum and maximum values, generating a two-column basis for each continuous variable. The exact positions of the three knots (internal and boundary) were derived exclusively from the training dataset and then held fixed for identical application to the validation dataset, thereby avoiding temporal information leakage.

For each participant, we obtained the predicted probability of normotension and of each phenotype from the fitted model. The overall probability of undiagnosed hypertension was defined as P(HTN) = 1 − P(normotension) = P(IDH) + P(ISH) + P(SDH), which is equivalent to summing the three phenotype-specific probabilities estimated by the multinomial model.

### 2.6. Temporal Validation

Temporal validation was performed by applying the model, with all coefficients and knot positions fixed, to the validation dataset (ENDES 2021–2024). Predictor variables were reconstructed in the validation dataset using identical coding, categorization, and exclusion criteria as in the training dataset. Natural cubic splines for age and BMI were evaluated on the validation data using knot positions derived from the training dataset—medians, minima, and maxima were not recalculated from the new dataset—thereby ensuring that the nonlinear transformation was strictly identical. No recalibration or retraining of any model coefficient was performed. This design reproduces the implementation scenario in which a model developed in one period is prospectively applied to future data without adjustments, thereby constituting a rigorous test of the temporal stability of the estimated coefficients.

### 2.7. Cutoff Selection and Performance Assessment

In the training dataset, an ROC curve was built for the binary composite outcome (any undiagnosed hypertension vs. normotension) using the predicted overall probability as the predictor. The operational cutoff was selected by maximizing the Youden index (J = sensitivity + specificity − 1), which identifies the threshold that yields the greatest separation between the true-positive rate and the false-positive rate. This cutoff was prespecified and applied without recalibration in the validation dataset, replicating the prospective implementation scenario in which thresholds are not re-tuned using future data. The Youden-derived cutoff should therefore be viewed as a pragmatic reference rather than a universal clinical threshold; in implementation, threshold choice should depend on local diagnostic confirmation capacity and the relative consequences of false negatives and false positives.

Discrimination was quantified using the area under the ROC curve (AUC), with 95% confidence intervals calculated using the DeLong method. We assessed overall discrimination for the binary composite outcome and for each phenotype using a one-versus-rest approach, in which each phenotype was defined as the positive case and the remainder of the sample as the negative case. Clinical performance at the prespecified cutoff was reported using sensitivity, specificity, positive predictive value (PPV), and negative predictive value (NPV), calculated from a confusion matrix weighted by the continuous sampling weight to reflect population proportions. To support implementation-oriented threshold selection, we also reported performance across a range of plausible probability cutoffs. Finally, we evaluated clinical utility using decision curve analysis and clinical impact curves in the validation cohort.

Calibration was evaluated in the validation dataset for both the binary composite outcome and each phenotype. Calibration plots were constructed by grouping participants into deciles of predicted probability and comparing the observed proportion (weighted mean) with the predicted proportion within each decile, with a superimposed loess curve. Three numeric calibration metrics were calculated: the weighted Brier score as a global measure of probabilistic accuracy, and the calibration intercept and slope obtained from a logistic regression of the observed outcome on the logit of the predicted probability (ideal values: intercept = 0, slope = 1). This evaluation was conducted separately for the overall outcome and for IDH, ISH, and SDH because a multinomial model may be well calibrated in aggregate yet display bias for specific phenotypes.

We explored equity in model performance across sex and residential altitude subgroups by reporting the AUC for the binary composite outcome in each stratum in the validation dataset. We did not refit or recalibrate separate models within subgroups; the same model coefficients were applied across all participants. Additionally, the numeric calibration metrics (Brier score, calibration intercept, and calibration slope) are summarized in the Results section.

### 2.8. Sampling Weights and Complex Survey Analysis

Descriptive estimates incorporated the complex ENDES design (strata: HV022; clusters: QHCLUSTER) and sampling weights. For the multiyear analysis, we constructed a pooled weight by dividing the original weight by the number of years in each dataset (weight_pooled = weight/n_years), so that the sum of weights would represent the average population size over the period. Descriptive tables report unweighted *n* and percentages based on this continuous weight with the full strata-and-cluster design.

To fit the multinomial model, we used frequency weights (weight_freq), obtained by rounding weight_pooled to the nearest integer. The multinom function from the nnet package does not natively support complex sample designs with strata and clusters; therefore, frequency weights allowed observations to contribute proportionally to the likelihood according to their population representativeness but did not account for clustering or stratification in variance estimation. Consequently, the ORs and confidence intervals from the model should be interpreted as weighted point estimates with independence-based standard errors rather than as estimates fully adjusted for the sample design. As a sensitivity analysis, we recomputed key classification metrics at the prespecified cutoff using the unrounded continuous sampling weights and obtained similar results. More design-aware estimation strategies (e.g., survey-weighted multinomial models or replication-based variance estimation) would further strengthen inference for individual coefficients, but are not required for the model’s intended predictive use and remain an area for future work.

The reported clinical performance metrics (sensitivity, specificity, PPV, and NPV) were calculated from confusion matrices weighted by the continuous pooled weight (weight_pooled), without rounding, to maintain consistency with the weighted prevalences in the descriptive tables. The AUC and its 95% confidence interval were estimated using the pROC package for the binary outcome, without weights, as a summary of ranking performance; weights were retained for descriptive estimates and prevalence-dependent performance metrics.

### 2.9. Software and Reproducibility

All analyses were performed in R (version 4.3; R Foundation for Statistical Computing, Vienna, Austria), using the survey package for complex sampling design, nnet package for multinomial regression, the splines package for natural cubic spline basis functions, and the pROC package for ROC curve analyses. Tables and figures were generated reproducibly from prespecified scripts.

### 2.10. Ethical Considerations

This study used publicly available, fully anonymized ENDES data and was therefore exempt from additional ethics review. ENDES obtained informed consent from participants at the time of data collection; no additional consent was required for this secondary analysis.

## 3. Results

### 3.1. Participant Selection

Of 170,937 records in the ENDES adult dataset, 74,719 participants were included in the training dataset (2017–2019) and 96,218 in the validation dataset (2021–2024). After excluding those who reported a prior hypertension diagnosis (6189 in training; 8075 in validation) and individuals younger than 18 years (3240 and 8307, respectively), we further excluded records with missing values in the outcome or any predictor, or with implausible BMI (3199/74,719 [4.3%] and 2464/96,218 [2.6%], respectively). The final analytic sample comprised 62,091 participants in training and 77,372 in validation (Figure S1).

### 3.2. Baseline Characteristics and Phenotype Distribution

Mean age was 40.2 years (SD 16.1) in the training dataset and 40.7 (SD 16.2) in the validation dataset; mean BMI was 27.1 (SD 4.6) and 27.4 (SD 5.0), respectively (Table 1). Both datasets were balanced by sex (49.9% women), and most participants resided at <1500 m altitude (75.1% in training; 75.4% in validation).

In the training dataset, 88.4% were classified as normotensive, 1.1% had IDH, 7.7% had ISH, and 2.8% had SDH (weighted percentages; unweighted *n* in Table 1). The weighted prevalence of any undiagnosed hypertension was 11.6%. In the validation dataset, 87.4% were normotensive, 3.0% had IDH, 5.0% had ISH, and 4.5% had SDH, with a weighted prevalence of undiagnosed hypertension of 12.6% (Table 1).

### 3.3. Comparison of Characteristics by Phenotype

In the training dataset (Table S2), participants with ISH and SDH were older on average than normotensive participants (55.3 [SD 18.6] vs. 48.9 [SD 14.0] vs. 38.6 [SD 15.2] years). BMI was higher in IDH and SDH (29.7 [SD 4.5] and 29.4 [SD 4.7], respectively) than in normotension (26.9 [SD 4.6]).

Sex distribution differed across phenotypes: men comprised 74.1% of IDH, 65.3% of ISH, and 79.0% of SDH. Differences were also observed in altitude distribution and in patterns of food and alcohol consumption across the four groups (Table S2).

### 3.4. Multinomial Model and Predictor Contribution

The multinomial model incorporated the eight prespecified nonlaboratory predictors, with nonlinear terms for age and BMI (Figure S2). Among categorical predictors, male sex showed consistently higher model-based odds of each phenotype (Table 2).

Male sex was associated with higher odds of IDH (OR 4.20; 95% CI 2.75–6.41), ISH (OR 2.17; 95% CI 1.90–2.48), and SDH (OR 4.14; 95% CI 3.27–5.25).

Compared with residence at <1500 m, residence at 1500–2499 m showed higher model-based odds of IDH (OR 2.63; 1.53–4.51) and lower model-based odds of ISH (OR 0.54; 0.40–0.74). Within the fitted model, risky alcohol consumption contributed to higher predicted odds of IDH (OR 2.35; 1.10–5.03), vegetable intake to higher predicted odds of ISH (OR 1.46; 1.19–1.79), and fruit intake to higher predicted odds of SDH (OR 1.64; 1.33–2.02) (Table 2). These coefficients reflect predictive contribution within the multivariable model and should not be interpreted as etiologic effects. Because age and BMI were modeled with splines, the interpretation of these continuous predictors is presented as marginal predicted probabilities (Figure S2) rather than as individual spline-basis coefficients.

### 3.5. Overall Model Performance

**Figure 1:**
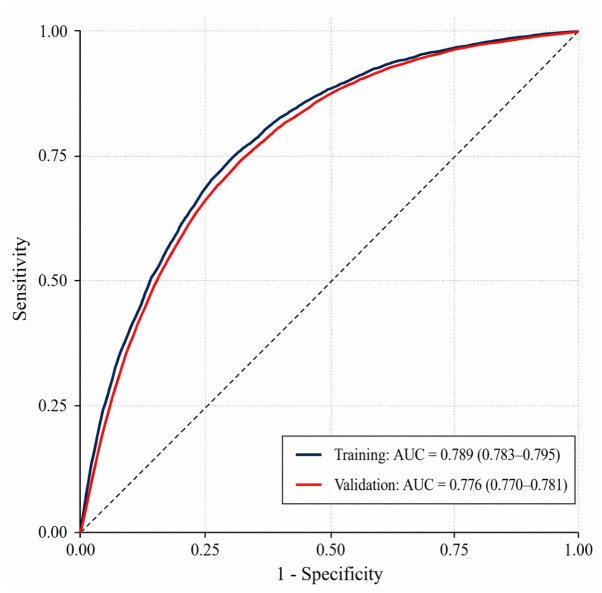
ROC curves for any undiagnosed hypertension in the training and temporal validation datasets. The dashed diagonal line represents random classification (AUC = 0.50). AUC values with 95% confidence intervals (DeLong method) are shown in the legend.

In the training dataset, the model achieved an AUC of 0.789 (95% CI, 0.783–0.795) for identifying any undiagnosed hypertension (Figure 1; Table 3). In temporal validation, the AUC was 0.776 (95% CI, 0.770–0.781), representing a reduction of 0.013 points compared with training (Figure 1; Table 3).

The cutoff derived from the training dataset (0.1004) was prespecified and held fixed during validation. At this threshold, sensitivity was 79.0% in training and 78.7% in validation; specificity was 63.2% and 60.9%, respectively. PPV was 22.0% in training and 22.5% in validation, while NPV was 95.8% and 95.2%, respectively (Table 3). Predicted probability distributions showed expected separation between normotension and undiagnosed hypertension and illustrate the proportion classified as high risk at the prespecified cutoff (Figure S3).

### 3.6. Performance by Phenotype and Calibration

In the validation dataset, phenotype discrimination was heterogeneous: AUC was 0.705 (95% CI, 0.694–0.716) for IDH, 0.817 (0.809–0.824) for ISH, and 0.788 (0.780–0.797) for SDH (Table S3; Figure S4). The lower AUC for IDH suggests a reduced ability to distinguish this phenotype from the rest of the sample.

**Figure 2:**
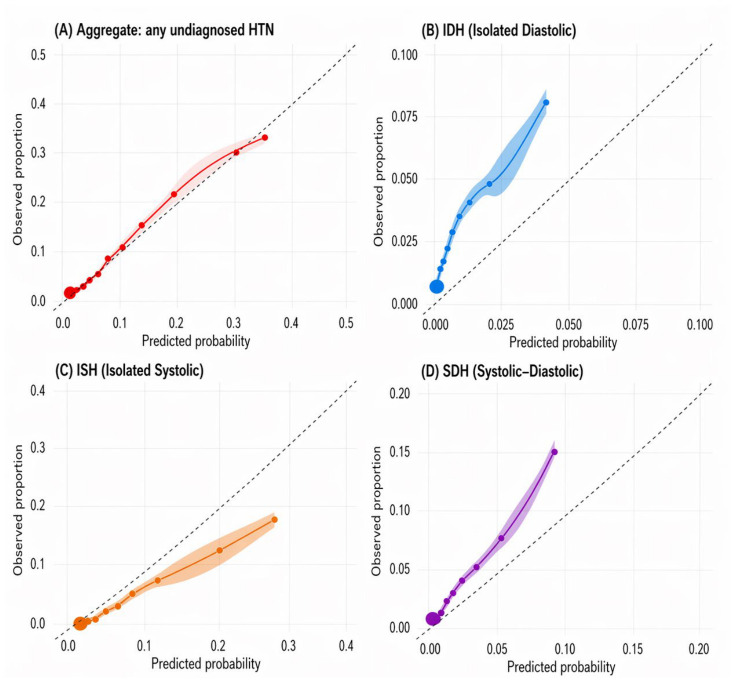
Calibration plots in the temporal validation dataset (ENDES 2021–2024): binary composite outcome and by individual phenotype. (**A**) calibration for any undiagnosed hypertension (binary). (**B**) IDH. (**C**) ISH. (**D**) SDH. Predicted probabilities were grouped into deciles. The dashed diagonal line represents perfect calibration. A loess smoother is superimposed. Colored solid lines represent the loess-smoothed calibration curves, shaded areas represent the corresponding uncertainty bands, and circles indicate observed outcome frequencies within deciles of predicted risk.

Overall calibration for the binary composite outcome in the validation dataset showed a Brier score of 0.0998, a calibration intercept of −0.100, and a calibration slope of 0.928 (Table S4; Figure 2A). Calibration by individual phenotype was heterogeneous: IDH had a slope of 0.577 and an intercept of −0.665; ISH had a slope of 0.996 and an intercept of −0.613; and SDH had a slope of 1.009 and an intercept of 0.476 (Table S4; Figure 2B–D). In particular, the IDH results indicate substantial miscalibration, suggesting that phenotype-specific IDH probabilities would require recalibration before operational use.

### 3.7. Subgroup Performance

In the validation dataset, AUC was relatively stable across altitude: 0.781 at <1500 m (*n* = 48,367), 0.780 at 1500–2499 m (*n* = 6317), 0.766 at 2500–3499 m (*n* = 14,318), and 0.766 at ≥3500 m (*n* = 8370). By sex, AUC was higher in women (0.786; *n* = 42,971) than in men (0.711; *n* = 34,401) (Figure S5). By age, discrimination decreased with increasing age (AUC 0.766 in 18–39 years; 0.703 in 40–59 years; and 0.610 in ≥60 years) (Table S5).

### 3.8. Threshold Analyses and Clinical Utility

Across alternative probability cutoffs in the validation cohort, the proportion classified as high risk ranged from 69.2% at a 0.05 threshold to 8.3% at a 0.30 threshold, with the expected sensitivity–specificity trade-off (Table S6). Decision curve analysis indicated a positive net benefit of using the model compared with “treat all” and “treat none” strategies across a clinically plausible range of threshold probabilities (Figure S6). In the clinical impact curve, the number classified as high risk and the expected number of true positives varied predictably with the threshold, supporting implementation planning (Figure S7). In a sensitivity analysis using continuous (unrounded) sampling weights, classification metrics at the prespecified cutoff were similar to those obtained using rounded frequency weights (Table S7).

## 4. Discussion

### 4.1. Main Findings

In this study, we developed and temporally validated a multinomial model based on eight nonlaboratory variables to identify undiagnosed hypertension and simultaneously classify three blood pressure phenotypes (IDH, ISH, and SDH) in Peruvian adults without a prior diagnosis. The model achieved an AUC of 0.789 in the training cohort (*n* = 62,091; ENDES 2017–2019) and 0.776 in temporal validation (*n* = 77,372; ENDES 2021–2024), with a reduction of only 0.013 points between cohorts. At a cutoff of 0.1004, sensitivity was 79.0% and 78.7%, and specificity was 63.2% and 60.9% in training and validation, respectively. Discrimination varied by phenotype, with higher values for ISH (AUC 0.817) and SDH (0.788) than for IDH (0.705). Overall calibration was acceptable (slope 0.928), but phenotype-specific calibration was notably weaker for IDH, suggesting that recalibration would be needed before phenotype-specific implementation. Beyond traditional performance metrics, decision curve and clinical impact analyses suggested that the model can offer positive net benefit across a range of plausible thresholds and achievable referral volumes for confirmatory measurement (Figures S6 and S7). Performance was stable across altitude but lower in men (AUC 0.711) than in women (0.786), which may reflect sex differences in phenotype mix, unmeasured determinants, or healthcare-contact patterns not captured by the model and warrants subgroup monitoring during implementation.

### 4.2. Comparison with Other Studies

The performance of our model (AUC 0.776 in temporal validation) falls within the range reported in the literature for cross-sectional diagnostic models aimed at detecting hypertension. A recent systematic review that specifically evaluated cross-sectional hypertension detection models found acceptable discrimination (AUC 0.70–0.89) and small differences between traditional statistical approaches and machine-learning methods, with consistently repeated predictors (age, BMI, and sex) and marginal gains from adding laboratory biomarkers (+0.02 to +0.04 in AUC) [11]. This evidence supports two methodological choices in our study: prioritizing a set of nonlaboratory predictors and using an interpretable, stable model (multinomial regression) rather than more complex algorithms whose incremental benefit may be limited relative to the implementation costs in primary care.

Large-scale studies using routine clinical data have reported higher AUCs. For example, Ji and colleagues, in a study of more than 4.2 million Chinese adults, found high performance of XGBoost using nonlaboratory variables (AUC 0.893) [20]. However, these differences likely reflect the massive sample size, the nature of the setting (health examinations), and the availability of predictors not captured in ENDES (e.g., clinical records). Moreover, many models are evaluated using internal validation or random splits; in contrast, our temporal validation approach—applying the same model to a later period with demographic and epidemiologic changes—is a more demanding standard for judging robustness and potential generalizability.

A distinctive feature of this work is the simultaneous prediction of undiagnosed hypertension phenotypes. Most existing tools collapse the outcome into a binary category, which may obscure clinical heterogeneity. Clinical evidence suggests that subtypes (e.g., IDH vs. ISH) differ in age distribution, mechanisms, and prognostic associations, with particular controversy surrounding IDH in young adults [6]. Because our model is intended for prediction rather than causal inference, the coefficients should be interpreted as conditional predictive contributions within the multivariable model—not as etiologic effects—and this is especially important for behavioral variables such as diet or alcohol use. This caution helps avoid the Table 2 fallacy [21], while still underscoring that phenotype-specific differences in discrimination and calibration may matter for screening and planning.

In the Peruvian context, this contribution is particularly relevant. National evidence shows that only about half of people with hypertension have a prior diagnosis [10,16], and that awareness, treatment, and control are particularly low in socioeconomically disadvantaged subgroups and rural areas [10]. In this setting, a tool based on interviews and basic anthropometry—without laboratory tests—could facilitate community strategies to prioritize whom to measure first or more frequently, especially when screening resources are insufficient for sustained universal coverage.

### 4.3. Public Health Implications

From a public health perspective, distinguishing blood pressure phenotypes may still be useful because subtypes differ in age distribution, pathophysiologic mechanisms, and prognostic associations, with particular controversy surrounding IDH in young adults [17,18]. At the same time, the model’s most immediate application is as a prescreening or triage tool before confirmatory blood pressure measurement. At the prespecified cutoff (0.1004), PPV was modest (~22%), implying a substantial number of false positives; however, this may be acceptable when the downstream action is low risk and inexpensive, namely standardized blood pressure measurement and confirmation. By contrast, the high NPV (95.2%) allows a large subgroup to be classified as low risk at that moment. Table S6 shows how these trade-offs change across alternative thresholds, and decision curve and clinical impact analyses help translate threshold choices into expected net benefit and referral volumes (Figures S6 and S7). In the Peruvian context, where prior diagnosis, treatment, and control remain incomplete [10], a tool based on interviews and basic anthropometry may help prioritize whom to measure first when screening resources are insufficient for sustained universal coverage.

The multinomial output adds a layer of information that may be useful for planning screening and health education. In practice, the phenotype-specific probability should not be interpreted as a diagnosis or as therapeutic guidance; however, it could alert healthcare teams to expected patterns. For example, a higher predicted risk of a predominantly diastolic phenotype could reinforce the need not to underestimate elevated diastolic values in young adults. In contrast, a profile compatible with ISH would emphasize the importance of systolic pressure at older ages [17,22]. The true clinical utility of this phenotypic stratification—beyond overall risk—requires further research and will depend on adequate calibration of class probabilities.

Finally, translation into practice requires implementation research. While the model relies on simple predictors, it includes spline terms for age and BMI; therefore, a programmed calculator (e.g., web or mobile) would be the most practical format for deployment rather than a paper checklist. In this study, we provide decision curve analysis, clinical impact curves, and performance across alternative thresholds (Table S6; Figures S6 and S7) to support implementation planning. Future studies should evaluate the feasibility, acceptability, costs, and real-world impact of opportunistic versus universal measurement strategies [9,23,24].

### 4.4. Limitations

This study should be interpreted in light of several limitations. First, ENDES is cross-sectional, and blood pressure is measured at a single visit (albeit with multiple readings), which creates a risk of misclassification (e.g., white-coat effect, biological variability, or masked hypertension). This bias is inherent to an interview- and survey-based tool and may affect both model development and validation. Second, because the multinomial algorithm did not fully accommodate the complex survey design, model fitting relied on frequency weights derived from sampling weights. At the same time, this may approximate population structure, and the standard errors and confidence intervals for coefficients may be underestimated. Third, we conducted a complete-case analysis. However, records were excluded because of missing data or implausible BMI, which represented a small proportion of eligible participants; we did not perform multiple imputation or report variable-specific missingness patterns, so selection bias cannot be excluded. Fourth, the model was developed and temporally validated only within ENDES Peru; temporal validation is valuable but does not replace geographic external validation in independent populations and healthcare systems. Fifth, phenotype-specific calibration, especially for IDH (slope 0.577; intercept −0.665), was suboptimal, so class-specific probabilities should be recalibrated before implementation. Sixth, several predictors (smoking, alcohol, and diet) are self-reported and susceptible to social desirability bias, which could affect performance in routine settings where the interview context differs from that of the survey. Seventh, some potentially informative predictors were unavailable or not harmonized across survey years, including family history of hypertension, physical activity, comorbidities such as diabetes, and richer measures of socioeconomic context. We explored discrimination by sex, altitude, and age strata (Figure S5; Table S5), but not by other potentially relevant strata such as rurality or region. Finally, although we assessed clinical utility using decision curve and clinical impact analyses and reported alternative operating thresholds, decision-analytic results remain sensitive to local referral capacity and opportunity costs, and we did not create a simplified points-based score for paper-based use.

## 5. Conclusions

In conclusion, we developed a multinomial model using eight nonlaboratory predictors to estimate the probability of undiagnosed hypertension and classify hemodynamic phenotypes in the Peruvian adult population. The model showed acceptable discrimination, temporal stability, and overall calibration, with weaker phenotype-specific calibration for IDH. Decision-analytic evaluation suggested potential clinical utility across a range of thresholds. Future work should prioritize geographic external validation, phenotype-specific recalibration (particularly for IDH), and implementation studies to assess feasibility and impact in routine care.

## Figures and Tables

**Table 1:** Baseline characteristics of the training (ENDES 2017–2019) and temporal validation (ENDES 2021–2024) datasets.

	Training Dataset	Validation Dataset
**Variable**	**Training (2017–2019)**1	**Validation (2021–2024)**1
Age	40.2 (16.1)	40.7 (16.2)
BMI	27.1 (4.6)	27.4 (5.0)
SBP	120.6 (16.3)	118.8 (16.5)
DBP	71.8 (9.7)	74.9 (10.2)
Sex		
Female	34,621 (49.9%)	42,971 (49.9%)
Male	27,470 (50.1%)	34,401 (50.1%)
Altitude		
<1500 m	39,102 (75.1%)	48,367 (75.4%)
1500–2499 m	5150 (6.3%)	6317 (5.6%)
2500–3499 m	10,878 (12.3%)	14,318 (13.2%)
≥3500 m	6961 (6.3%)	8370 (5.8%)
Smoking status		
Never smoker	50,456 (79.3%)	64,173 (82.1%)
Former smoker	4949 (8.7%)	5580 (7.7%)
Current smoker	5725 (10.3%)	6651 (8.7%)
Daily smoker	961 (1.8%)	968 (1.4%)
Alcohol consumption		
None	41,065 (62.3%)	51,010 (62.7%)
Moderate consumption	19,966 (35.5%)	24,820 (34.8%)
Risky consumption	1060 (2.2%)	1542 (2.5%)
Vegetable intake		
No	57,854 (92.0%)	71,449 (92.0%)
Yes	4237 (8.0%)	5923 (8.0%)
Fruit intake		
No	48,954 (77.5%)	61,470 (78.5%)
Yes	13,137 (22.5%)	15,902 (21.5%)
Phenotype		
Normotension	56,413 (88.4%)	70,206 (87.4%)
IDH	710 (1.1%)	1932 (3.0%)
ISH	3568 (7.7%)	2816 (5.0%)
SDH	1400 (2.8%)	2418 (4.5%)
1 Mean (SD); *n* (unweighted) (%)		

Continuous values are reported as mean (SD). Categorical variables are reported as unweighted *n* (weighted percentage). SD = standard deviation. BMI = body mass index. SBP = systolic blood pressure. DBP = diastolic blood pressure. IDH = isolated diastolic hypertension. ISH = isolated systolic hypertension. SDH = systolic–diastolic hypertension.

**Table 2:** Odds ratios (ORs) and 95% confidence intervals from the multinomial logistic regression model for phenotypes of undiagnosed hypertension (reference category: normotension).

Predictor	IDH	ISH	SDH
	OR (95% CI)	OR (95% CI)	OR (95% CI)
Sex (ref: Female)			
Male	4.20 (2.75–6.41)	2.17 (1.90–2.48)	4.14 (3.27–5.25)
Altitude (ref: <1500 m)			
1500–2499 m	2.63 (1.53–4.51)	0.54 (0.40–0.74)	0.82 (0.52–1.28)
2500–3499 m	1.27 (0.69–2.33)	0.70 (0.55–0.88)	0.91 (0.64–1.30)
≥3500 m	1.36 (0.55–3.39)	0.76 (0.52–1.10)	0.96 (0.55–1.67)
Smoking status (ref: Never smoker)			
Former smoker	0.89 (0.52–1.53)	0.82 (0.64–1.05)	1.28 (0.94–1.73)
Current smoker	1.08 (0.68–1.71)	1.21 (0.99–1.48)	1.06 (0.78–1.43)
Daily smoker	0.89 (0.32–2.47)	0.66 (0.42–1.05)	0.84 (0.46–1.53)
Alcohol consumption (ref: None)			
Moderate	1.19 (0.84–1.69)	1.05 (0.92–1.21)	1.14 (0.93–1.40)
Risky	2.35 (1.10–5.03)	1.11 (0.75–1.64)	1.60 (0.97–2.63)
Vegetable intake (ref: No)			
Yes	0.65 (0.33–1.28)	1.46 (1.19–1.79)	1.12 (0.80–1.56)
Fruit intake (ref: No)			
Yes	1.03 (0.69–1.52)	1.14 (0.99–1.32)	1.64 (1.33–2.02)

Age and BMI were modeled using natural cubic splines (one internal knot at the median); coefficients of spline basis functions should not be interpreted as linear ORs. Interpretation of these continuous effects is presented graphically in Figure S2.

**Table 3:** Model performance for identifying any undiagnosed hypertension (IDH, ISH, or SDH) versus normotension in the training and temporal validation datasets.

Dataset	*n* (Unweighted)	Weighted Prevalence (%)	AUC (95% CI)	Sensitivity (%)	Specificity (%)	PPV (%)	NPV (%)	Optimal Cutoff
Training (2017–2019)	62,091	11.6	0.789 (0.783–0.795)	79.0	63.2	22.0	95.8	0.1004
Validation (2021–2024)	77,372	12.6	0.776 (0.770–0.781)	78.7	60.9	22.5	95.2	0.1004

AUC = area under the ROC curve (95% confidence interval by the DeLong method). PPV = positive predictive value. NPV = negative predictive value. The cutoff corresponds to the threshold selected by the Youden index in the training dataset.

## Data Availability

The data presented in this study are available in the INEI microdata repository at https://proyectos.inei.gob.pe/microdatos/ (accessed on 17 April 2026). These data were derived from the following public-domain resource: ENDES Peru 2017–2024.

## Supplementary Materials

The following supporting information can be downloaded at: https://www.mdpi.com/article/10.3390/medsci14020224/s1, Figure S1: Participant selection flow diagram; Figure S2: Nonlinear effects of age and BMI modeled with restricted cubic splines; Figure S3: Predicted probability distributions by case status at the prespecified cutoff; Figure S4: ROC curves by individual hypertension phenotype (one-vs-rest); Figure S5: Subgroup discrimination by altitude and sex; Figure S6: Decision curve analysis; Figure S7: Clinical impact curve; Table S1: TRIPOD + AI checklist; Table S2: Participant characteristics by phenotype (training cohort); Table S3: Discrimination by phenotype (validation cohort); Table S4: Calibration metrics by phenotype; Table S5: AUC by age strata; Table S6: Classification performance across alternative thresholds; Table S7: Sensitivity analysis using continuous sampling weights.
