# Supplementary material for: Development and Temporal Validation of a Multinomial Prediction Model for Phenotypes of Undiagnosed Hypertension in Peru: A Population-Based Study
Source: Med Sci (Basel). 2026 Apr 29;14(2):224. doi: 10.3390/medsci14020224 (PMC13214680; doi:10.3390/medsci14020224)
Supplement: Supplementary file 1 [file medsci-14-00224-s001.zip › medsci-4213861-supplementary.pdf]

Supplementary Materials: Development and Temporal Validation of a Multinomial Prediction Model for Phenotypes of Undiagnosed Hypertension in Peru: A Population-Based Study

Víctor Juan Vera-Ponce 1,*, Jhosmer Ballena-Caicedo 1, Holly Estrella Delgado-Toro 1, Fiorella E. Zuzunaga-Montoya 1, Julio César Bautista Zuta 1,2, Rossmery Leonor Poemape Mestanza 1,2

1 Facultad de Medicina (FAMED), Universidad Nacional Toribio Rodríguez de Mendoza de Amazonas (UNTRM), Amazonas, Perú
2 Hospital Regional Virgen de Fátima, Chachapoyas, Peru
* Correspondence: victor.vera@untrm.edu.pe; Tel.: +51-957061401

Table S1. TRIPOD+AI 2024 Checklist. Prediction model development and evaluation study. D = development; E = evaluation.

Section/Topic	Item	Checklist requirement	Location in manuscript
**TITLE**			
Title	1 (D;E)	Identify the study as development or evaluation of a multivariable prediction model, the target population, and the predicted outcome	Title, p. 1
**ABSTRACT**			
Abstract	2 (D;E)	Structured abstract according to the TRIPOD+AI checklist for abstracts	Abstract, p. 2
**INTRODUCTION**			
Background	3a (D;E)	Explain the clinical context (diagnostic or prognostic) and the rationale for developing or evaluating the model, including references to existing models	Introduction, paragraphs 1–4
	3b (D;E)	Describe the target population and the intended purpose of the model within the care pathway, including intended users	Introduction, paragraphs 5–6; also Discussion, "Public health implications"
	3c (D;E)	Describe known health inequalities between sociodemographic groups	Introduction, paragraph 3 (gaps in diagnosis, treatment, and control by socioeconomic status and place of residence)
Objectives	4 (D;E)	Specify the study objectives, including whether it describes model	Introduction, last paragraph ("the aim of this study was to develop and temporally validate…")

		development or evaluation (or both)	
**METHODS**			
Datos	5a (D;E)	Describe the data sources separately for development and evaluation, the rationale for their use, and representativeness	Methods, "Study design and data source" and "Data source"
	5b (D;E)	Specify the dates of participant data collection	Methods, "Datasets and eligibility criteria" (training: 2017–2019; validation: 2021–2024)
Participantes	6a (D;E)	Specify key elements of the study setting, including the number and location of centers	Methods, "Data source" (national survey; 24 departments and Callao; urban and rural areas)
	6b (D;E)	Describe participant eligibility criteria	Methods, "Datasets and eligibility criteria" (four exclusion criteria)
	6c (D;E)	Provide details of treatments received, if relevant	Not applicable (cross-sectional diagnostic model; individuals with a prior hypertension diagnosis were excluded)
Data preparation	7 (D;E)	Describe data preprocessing and quality control procedures	Methods, "Blood pressure measurement" (implausible BP filters), "Candidate predictors" (implausible BMI filter <10 or ≥60; recoding missing fruit/vegetable intake as zero portions)
Desenlace	8a (D;E)	Clearly define the predicted outcome, including how and when it was assessed, and its consistency across sociodemographic groups	Methods, "Blood pressure measurement and outcome definition" (four categories with a 140/90 mmHg threshold; threshold justification; binary composite outcome)
	8b (D;E)	If outcome assessment requires subjective interpretation, describe the assessor qualifications	Not applicable (objective measurement with a digital OMRON HEM-713 sphygmomanometer; standardized protocol)
	8c (D;E)	Report any actions to blind outcome assessment to predictors	Methods, “Blood pressure measurement” (standardized measurement by trained staff following the ENDES protocol; self-reported predictors are collected independently of BP measurement)

Predictores	9a (D)	Describe initial predictor selection and any preselection before model building	Methods, "Candidate predictors", paragraph 1 (prespecified for clinical plausibility and feasibility) and paragraph 4 (rationale for excluding ethnicity and wealth index)
	9b (D;E)	Clearly define all predictors, including how and when they were measured	Methods, "Candidate predictors", paragraphs 2–3 (operational definition of 8 variables with ENDES codes: HV104, HV040, QS700–QS707, QS600–QS614, QS214C, QS220CV)
	9c (D;E)	If predictor measurement requires subjective interpretation, describe the assessor qualifications	Not applicable (objective variables: anthropometry with calibrated equipment; categorical variables self-reported via a standardized questionnaire)
Sample size	10 (D;E)	Explain how the sample size was determined and justify that it was sufficient	Methods/Results: all available ENDES data were used. Training: n=62,091; validation: n=77,372. No formal sample size calculation was performed; the size of both datasets far exceeds recommendations for models with 8 predictors
Datos faltantes	11 (D;E)	Describe how missing data were handled	Methods, “Datasets and eligibility criteria” (complete-case analysis; exclusion of records with missing data); “Candidate predictors” (missing fruit/vegetable values recoded as zero portions); Limitations, paragraph on complete-case analysis
Analytical methods	12a (D)	Describe how the data were used in the analysis, including any partitioning	Methods, "Datasets and eligibility criteria" (two temporally independent datasets: 2017–2019 for development, 2021–2024 for temporal evaluation; no random split)
	12b (D)	Describe how predictors were handled in the analysis (functional form, transformations)	Methods, "Model development", paragraph 3 (natural cubic splines with 2 df for age and BMI; knots

			fixed from training); categorical variables without transformation
	12c (D)	Specify the type of model, rationale, model-building steps, and internal validation method	Methods, "Model development" (multinomial logistic regression, nnet::multinom, 4 categories, 3 logit equations, max. 500 iterations; no variable selection; frequency weights); internal validation was not formally performed (temporal external validation was chosen instead)
	12d (D;E)	Describe whether and how heterogeneity between clusters was handled	Methods, "Sampling weights" (ENDES complex design with strata HV022 and clusters QHCLUSTER; nnet limitations explicitly described); Results, "Subgroup performance" (AUC by altitude and sex)
	12e (D;E)	Specify all measures and plots used to assess model performance	Methods, "Cutoff selection and performance assessment" (AUC with 95% CI by DeLong; sensitivity, specificity, PPV, NPV; weighted Brier score; calibration intercept and slope; calibration plots with loess; AUC by subgroups)
	12f (E)	Describe any model updating after evaluation	Methods, "Temporal external validation" (no recalibration or retraining was performed); Conclusions (future recalibration is recommended)
	12g (E)	For model evaluation, describe how predictions were calculated	Methods, "Temporal external validation" (coefficients and spline knots fixed from training; variables reconstructed using identical criteria; P(HTN) = 1 − P(normotension))
Class imbalance	13 (D;E)	If methods to correct class imbalance were used, indicate why and how	No class imbalance correction methods were applied
Equidad	14 (D;E)	Describe any approach to address model equity and its rationale	Methods, "Cutoff selection" (AUC by sex and altitude); Results, "Subgroup

			performance"; Discussion, "Implications" (lower discrimination in men; recommendation for local monitoring and recalibration)
Model output	15 (D)	Specify the model output (probabilities, classification) and details of any cutoff	Methods, "Model development" (four mutually exclusive probabilities); "Cutoff selection" (cutoff 0.1004 by Youden index; note that in implementation the threshold should be adapted to local capacity)
Development vs. evaluation	16 (D;E)	Identify differences between development and evaluation data	Results, "Baseline characteristics" (Table 1 compares both datasets); Methods, "Temporal external validation" (identical coding and exclusion criteria)
Ethics approval	17 (D;E)	Name the ethics committee that approved the study	Methods, "Ethical considerations" (secondary analysis of anonymized public-access data; ethics approval was not required)
**CIENCIA ABIERTA**			
Financiamiento	18a (D;E)	Provide the funding source and the role of the funders	The study was self-funded
Conflicts of interest	18b (D;E)	Declare conflicts of interest for all authors	The authors declare no conflicts of interest
Protocolo	18c (D;E)	Indicate where the study protocol can be accessed	No protocol was prepared in advance
Registro	18d (D;E)	Provide study registration information	The study was not previously registered
Datos compartidos	18e (D;E)	Provide details on data availability	ENDES microdata are publicly available through the INEI portal (https://www.inei.gob.pe)
Code sharing	18f (D;E)	Provide details on the availability of the analytical code	Methods, "Software and reproducibility" (R 4.3; survey, nnet, splines, pROC packages). [Pending: consider depositing the code in a public repository]
**PATIENT AND PUBLIC INVOLVEMENT**			
PPI	19 (D;E)	Provide details of patient and public involvement, or state that there was none	There was no patient or public involvement in the design, conduct, or reporting of the study
**RESULTADOS**			

Participantes	20a (D;E)	Describe the flow of participants, including the numbers with and without the outcome	Results, "Participant selection"; Figure S1 (flow diagram)
	20b (D;E)	Report participant characteristics overall and by data source, including missing data	Results, "Baseline characteristics" (Table 1); "Comparison by phenotype" (Table S1)
	20c (E)	For model evaluation, show comparison with development data for the distribution of important predictors	Table 1 (compares training vs validation)
Model development	21 (D;E)	Specify the number of participants and events in each analysis	Results: training n=62,091 (undiagnosed hypertension: 5,678 cases, 11.6%); validation n=77,372 (7,166 cases, 12.5%)
	22 (D)	Present the full model to allow individual predictions (coefficients, intercepts)	Table 2 (ORs and 95% CIs for categorical predictors); Figure S5 (spline effects).
Model performance	23 (D;E)	Report performance measures with confidence intervals for the model	Results, "Overall performance" (Table 3: AUC, sensitivity, specificity, PPV, NPV); "Performance by phenotype" (Table S2: phenotype-specific AUC); "Calibration" (Table S3: Brier score, intercept, slope; Figure 2)
**DISCUSSION**			
Limitations	24 (D;E)	Discuss study limitations	Discussion, "Limitations" (seven explicit limitations)
Interpretation	25a (E)	For model evaluation, discuss the results in relation to performance in development data and other studies	Discussion, "Main findings" and "Comparison with other studies"
	25b (D;E)	Provide an overall interpretation considering objectives, limitations, similar studies, and other relevant evidence	Discussion, "Public health implications" and "Conclusions and recommendations"
Implications	26 (D;E)	Discuss the potential clinical use of the model and implications for future research	Discussion, "Public health implications" (prioritization, calculator, HEARTS); "Conclusions" (three recommendations: external validation, recalibration, implementation studies)

Table S2. Participant characteristics by blood pressure phenotype in the training dataset.

Variable	Overall n = 62,0911	Normotension n = 56,4131	IDH n = 7101	ISH n = 3,5681	SDH n = 1,4001
**Age**	40.2 (16.1)	38.6 (15.2)	40.1 (11.0)	55.3 (18.6)	48.9 (14.0)
**BMI**	27.1 (4.6)	26.9 (4.6)	29.7 (4.5)	28.2 (4.8)	29.4 (4.7)
**SBP**	120.6 (16.3)	116.5 (11.6)	132.9 (5.8)	150.9 (11.1)	159.2 (16.2)
**DBP**	71.8 (9.7)	70.2 (8.2)	92.9 (2.8)	79.2 (7.2)	96.3 (5.9)
**Sex**					
Female	34,621 (49.9%)	32,713 (52.4%)	257 (25.9%)	1,307 (34.7%)	344 (21.0%)
Male	27,470 (50.1%)	23,700 (47.6%)	453 (74.1%)	2,261 (65.3%)	1,056 (79.0%)
**Altitude**					
<1500 m	39,102 (75.1%)	35,469 (74.5%)	414 (71.4%)	2,308 (80.5%)	911 (79.1%)
1500–2499 m	5,150 (6.3%)	4,647 (6.4%)	71 (8.9%)	296 (5.4%)	136 (6.0%)
2500–3499 m	10,878 (12.3%)	9,909 (12.6%)	135 (13.2%)	600 (9.5%)	234 (10.3%)
≥3500 m	6,961 (6.3%)	6,388 (6.5%)	90 (6.6%)	364 (4.7%)	119 (4.6%)
**Smoking status**					
Never smoker	50,456 (79.3%)	46,025 (79.4%)	540 (72.3%)	2,892 (81.4%)	999 (72.3%)
Former smoker	4,949 (8.7%)	4,477 (8.8%)	72 (11.1%)	249 (6.3%)	151 (11.7%)
Current smoker	5,725 (10.3%)	5,097 (10.1%)	83 (14.3%)	349 (10.6%)	196 (13.4%)
Daily smoker	961 (1.8%)	814 (1.8%)	15 (2.3%)	78 (1.8%)	54 (2.7%)
**Alcohol consumption**					
None	41,065 (62.3%)	37,524 (62.7%)	376 (48.0%)	2,388 (63.5%)	777 (52.6%)
Moderate consumption	19,966 (35.5%)	17,963 (35.2%)	312 (48.2%)	1,113 (34.4%)	578 (43.8%)
Risky consumption	1,060 (2.2%)	926 (2.1%)	22 (3.8%)	67 (2.1%)	45 (3.6%)
**Vegetable intake**					
No	57,854 (92.0%)	52,543 (92.1%)	656 (92.8%)	3,344 (91.3%)	1,311 (91.7%)
Yes	4,237 (8.0%)	3,870 (7.9%)	54 (7.2%)	224 (8.7%)	89 (8.3%)
**Fruit intake**					
No	48,954 (77.5%)	44,469 (77.5%)	549 (76.7%)	2,855 (78.7%)	1,081 (72.6%)
Yes	13,137 (22.5%)	11,944 (22.5%)	161 (23.3%)	713 (21.3%)	319 (27.4%)

Continuous values are reported as mean (SD). Categorical variables are reported as unweighted n (weighted percentage). This table is descriptive; p-values are not reported because the objective of the study is prediction, not association inference.

Table S3. Outcome calibration in the temporal validation dataset.

Outcome	Brier_score	Cal_intercept	Cal_slope
Global	0.0998	-0.100	0.928
IDH	0.0293	-0.665	0.577
ISH	0.0467	-0.613	0.996
SDH	0.0415	0.476	1.009

The Brier score was calculated as the weighted mean of $(p - y)^2$. The calibration intercept and slope were obtained from a weighted logistic regression of the observed outcome on the logit of the predicted probability (ideal values: intercept = 0, slope = 1).

Table S4. Discrimination by individual phenotype (one-versus-rest approach) in the temporal validation dataset.

**Phenotype**	**AUC (95% CI)**
IDH	0.705 (0.694-0.716)
ISH	0.817 (0.809-0.824)
SDH	0.788 (0.780-0.797)

AUC = area under the ROC curve with 95% confidence intervals. Each phenotype was evaluated as the positive case versus the rest of the sample.

Table S5. Diagnostic performance across alternative risk thresholds (Validation cohort, ENDES 2021–2024).

**Threshold**	**High-risk (%)**	**Sensitivity (%)**	**Specificity (%)**	**PPV (%)**	**NPV (%)**
0.050	69.2	93.4	34.3	17.0	97.3
0.075	54.9	86.2	49.6	19.7	96.1
0.1004	44.0	78.7	60.9	22.5	95.2
0.125	35.7	70.9	69.3	25.0	94.3
0.150	28.9	62.3	75.9	27.1	93.3
0.175	23.7	54.8	80.8	29.0	92.6
0.200	19.2	47.1	84.8	30.8	91.8
0.250	12.6	34.1	90.5	34.1	90.5
0.300	8.3	23.6	93.9	35.6	89.5

Estimates are weighted using the pooled ENDES sampling weight (peso_pooled). High-risk is defined as predicted probability ≥ threshold. PPV and NPV depend on outcome prevalence in the target population.

Table S6. Discrimination (AUC) by age strata for the composite outcome (Validation cohort, ENDES 2021–2024).

**Age group**	**n (unweighted)**	**AUC**
18-39	48,111	0.766
40-59	20,237	0.703
≥60	9,027	0.610

AUCs are unweighted and based on the composite outcome (any undiagnosed HTN) vs normotension.

Table S7. Sensitivity analysis of weighting approach for classification metrics at the prespecified cutoff (0.1004).

**Cohort**	**Weighting approach**	**Prevalence (%)**	**Sensitivity (%)**	**Specificity (%)**	**PPV (%)**	**NPV (%)**

Training (ENDES 2017–2019)	Continuous sampling weight (peso_pooled)	11.6	79.0	63.2	22.0	95.8
Training (ENDES 2017–2019)	Rounded frequency weight (peso_freq)	13.2	81.1	58.7	22.9	95.3
Validation (ENDES 2021–2024)	Continuous sampling weight (peso_pooled)	12.6	78.7	60.9	22.5	95.2
Validation (ENDES 2021–2024)	Rounded frequency weight (peso_freq)	15.0	81.1	55.9	24.5	94.4

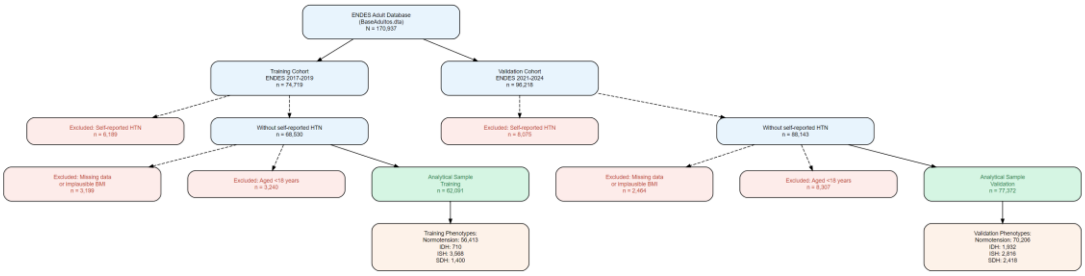

Figure S1. Participant selection flow diagram. ENDES = Demographic and Family Health Survey. HTN = hypertension. IDH = isolated diastolic hypertension. ISH = isolated systolic hypertension. SDH = systolic-diastolic hypertension.

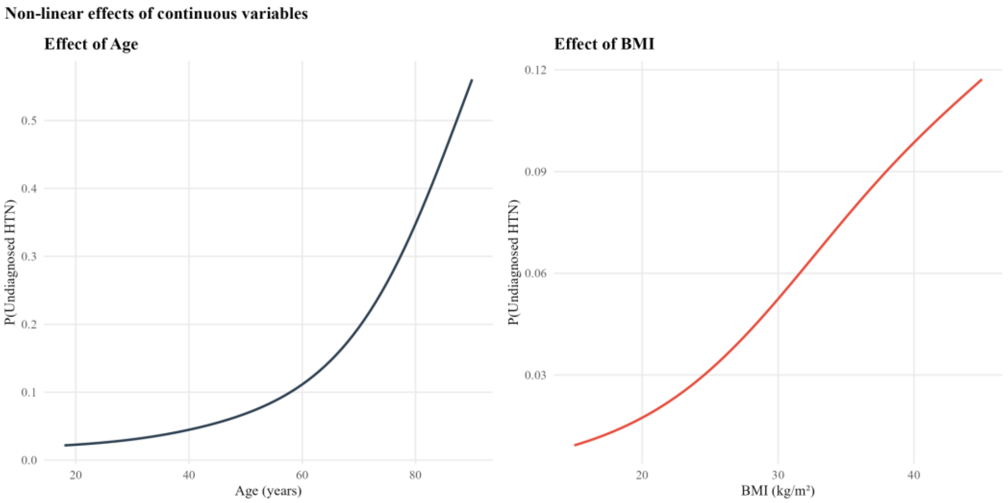

Figure S2. Nonlinear effects of age and BMI on the predicted probability of undiagnosed hypertension. Marginal predicted probabilities derived from the multinomial model, varying one variable at a time while holding the others at their modal or median values.

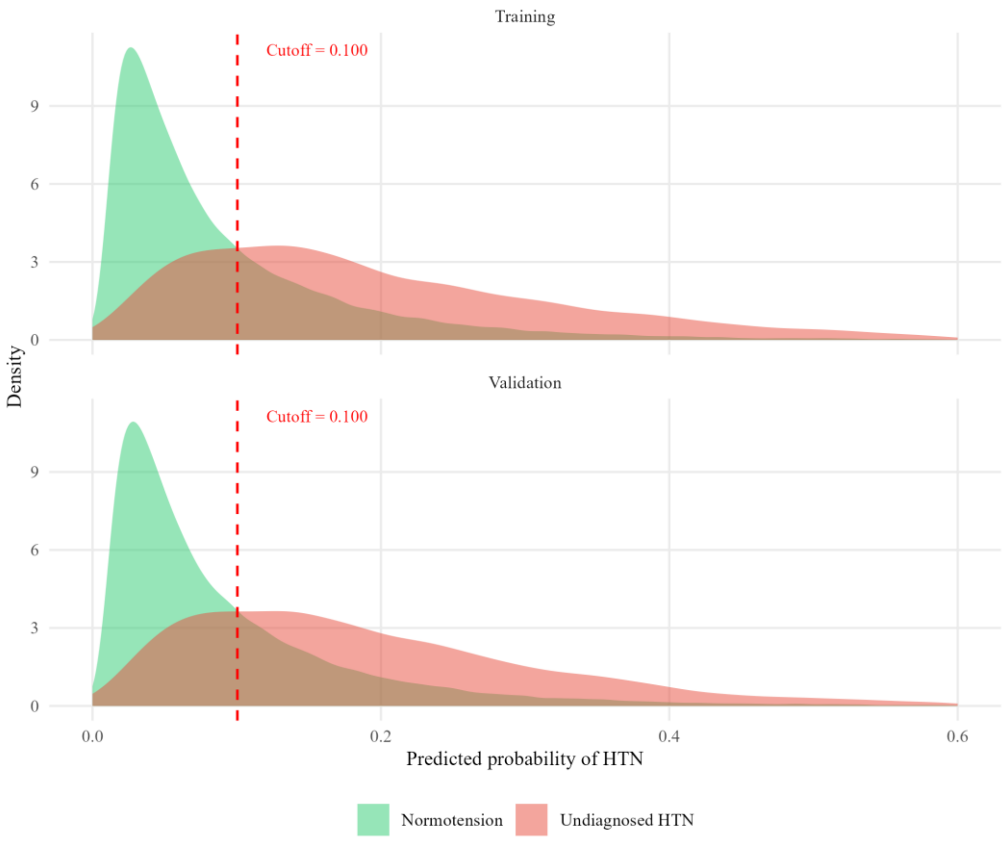

Figure S3. Distribution of predicted probabilities of undiagnosed hypertension by true status in the training and validation datasets. The dashed red vertical line indicates the optimal cutoff derived by the Youden index in the training dataset.

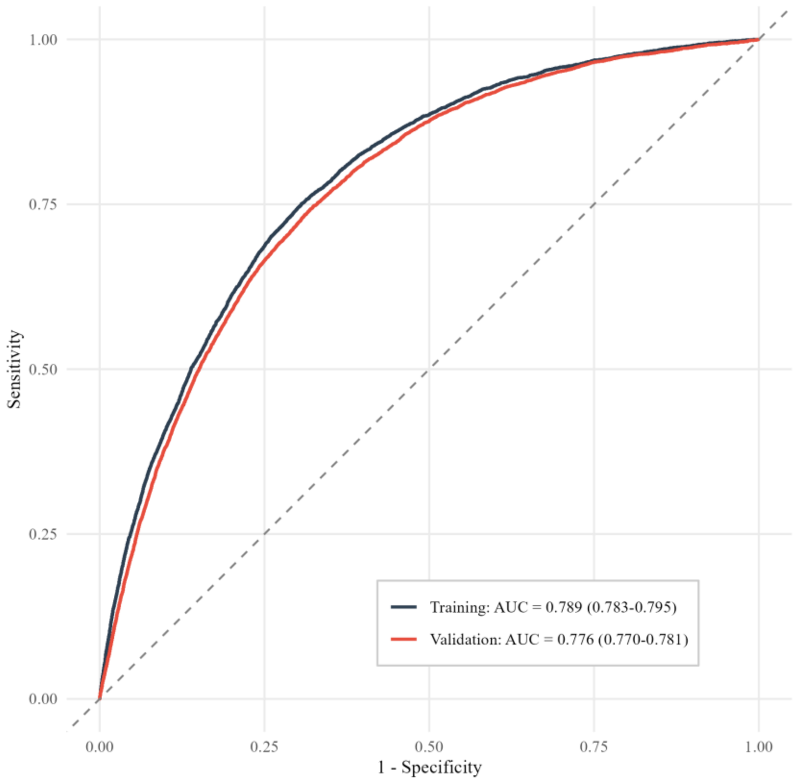

Figure S4. ROC curves by individual phenotype (one-versus-rest approach) in the temporal validation dataset (ENDES 2021–2024). The ROC curve and corresponding AUC are shown for each phenotype evaluated individually versus the rest of the sample.

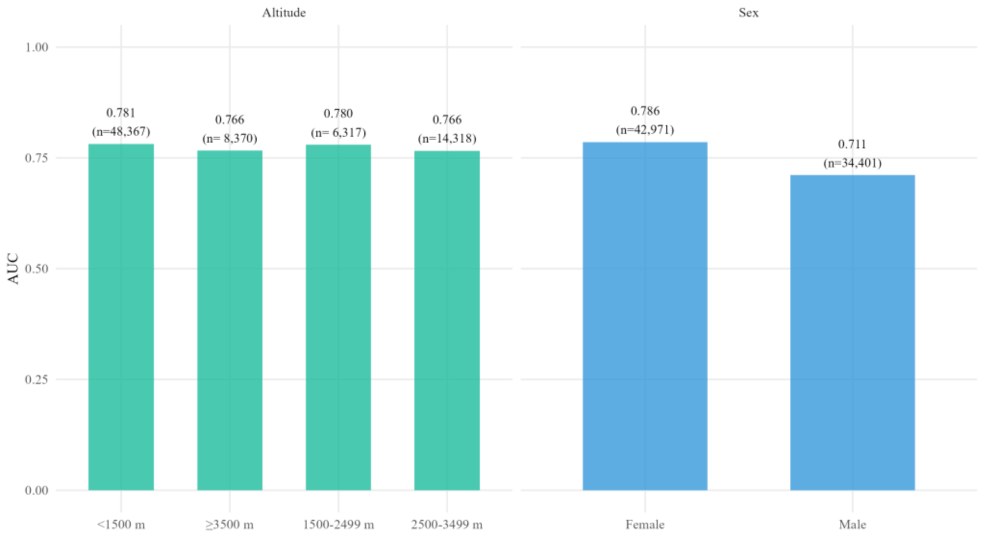

Figure S5. Model AUC in subgroups by sex and residential altitude in the temporal validation dataset. The AUC for the binary composite outcome is shown stratified by sex and altitude category.

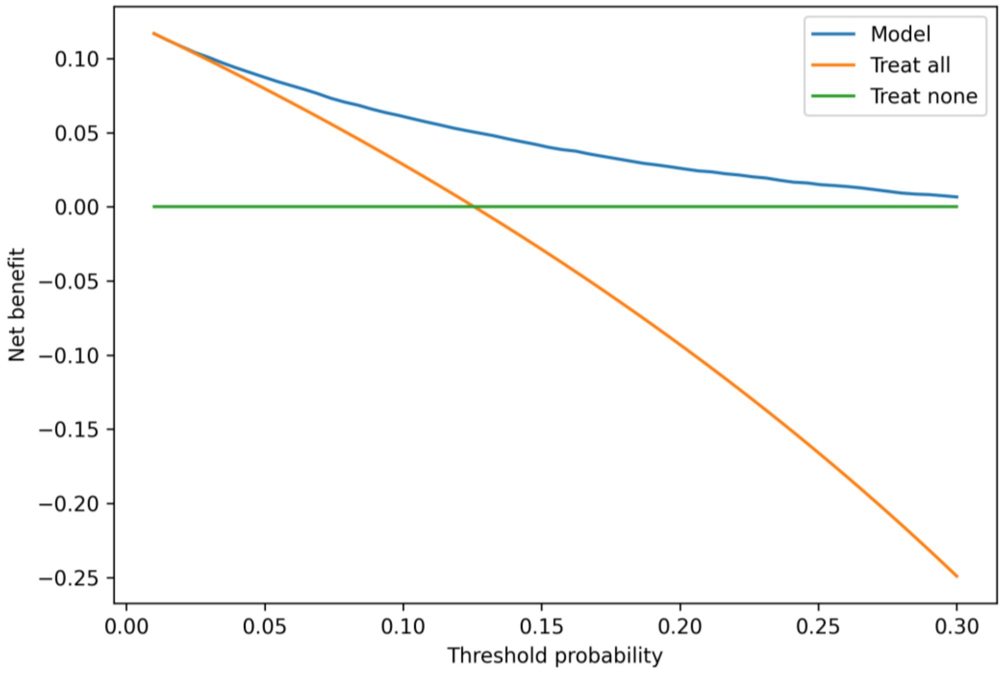

Figure S6. Decision curve analysis (validation cohort).

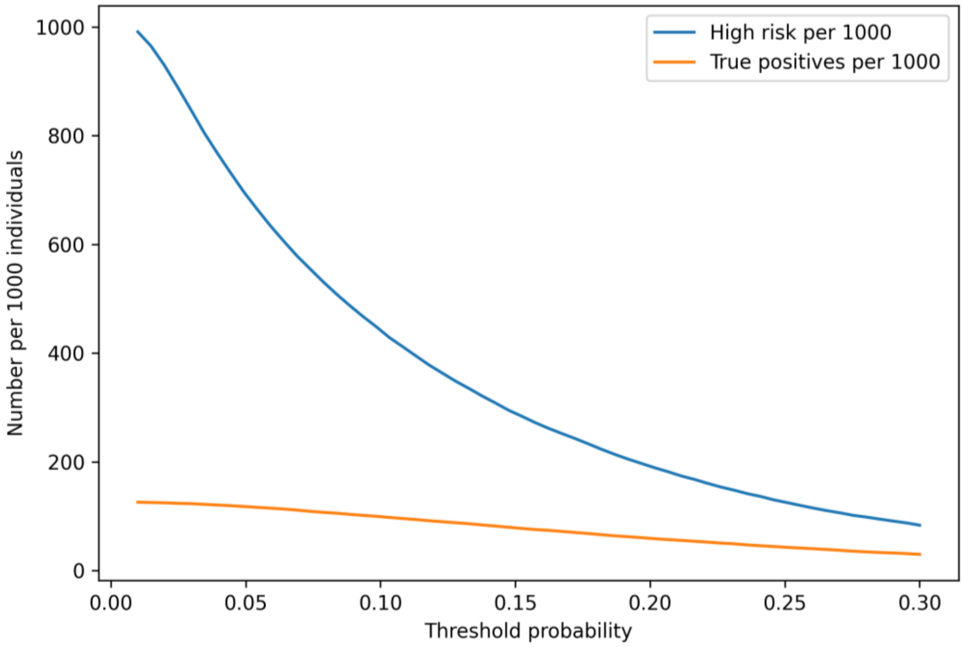

Figure S7. Clinical impact curve (validation cohort)
